# Epidemiology and Risk Factors for Carbapenem-Resistant *Klebsiella Pneumoniae* and Subsequent MALDI-TOF MS as a Tool to Cluster KPC-2-Producing *Klebsiella Pneumoniae*, a Retrospective Study

**DOI:** 10.3389/fcimb.2020.00462

**Published:** 2020-09-14

**Authors:** Lili Fang, Heping Xu, Xiaoying Ren, Xun Li, Xiaobo Ma, Haijian Zhou, Guolin Hong, Xianming Liang

**Affiliations:** ^1^Department of Clinical Laboratory, The First Affiliated Hospital, School of Medicine, Xiamen University, Xiamen, China; ^2^Xiamen Key Laboratory of Genetic Testing, Xiamen, China; ^3^School of Public Health, Xiamen University, Xiamen, China; ^4^State Key Laboratory for Infectious Disease Prevention and Control, Chinese Center for Disease Control and Prevention, National Institute for Communicable Disease Control and Prevention, Beijing, China; ^5^Collaborative Innovation Center for Diagnosis and Treatment of Infectious Diseases, Hangzhou, China; ^6^Center of Clinical Laboratory, School of Medicine, Zhongshan Hospital, Xiamen University, Xiamen, China; ^7^Institute of Infectious Disease, School of Medicine, Xiamen University, Xiamen, China

**Keywords:** carbapenems, resistance, *Klebsiella pneumonia*, carbapenem-resistant *Klebsiella pneumonia*, MALDI-TOF MS

## Abstract

**Background:** Carbapenem-resistant *Klebsiella pneumoniae* (CRKP) appeared recently and now presents a particularly critical problem to hospitalized patients worldwide. We aim to investigate the epidemiology and the risk factors for CRKP colonization and infections, and to evaluate the application performance of MALDI-TOF MS in clustering CRKP.

**Results:** CRKP colonization and infections incidence was 2.7 (35/1,319,427) per 100,000 patient-days. Inpatients in CRKP group had higher medical expense than CSKP group. Inpatients with underlying conditions, particularly with pulmonary diseases, and with antimicrobial use prior to culture within 30 days, especially with carbapenem use, were risk factors for CRKP acquisition. All CRKP isolates were detected producing KPC-2. The MALDI-TOF MS system and PFGE system provided similar results, with a good concordance between the two methods (adjusted Rand's coefficient, 0.846) and a high probability of MALDI-TOF MS to predict PFGE results (Wallace coefficient, 0.908).

**Conclusions:** Underlying conditions, particularly pulmonary diseases, and antimicrobial use prior to culture within 30 days, especially carbapenem use, are risk factors for CRKP acquisition. *Bla*_*KPC*−2_ is the mainstream gene of CRKP in our geographic area of analysis. As only simple sample preparation is needed and the results can be obtained in a short time, MALDI-TOF MS may be considered a probable alternative to PFGE in clustering KPC-2-producing CRKP.

## Background

Carbapenems are widely used due to their broad spectrum of activity. Nevertheless, carbapenem-resistant *Klebsiella pneumoniae* (CRKP) appeared and now presents a particularly critical problem to hospitalized patients worldwide (Yigit et al., 2001; Canton et al., 2012; McConville et al., 2017; Asai et al., 2018). The limited clinical options often make anti-infective therapy extremely difficult and also cause an extra financial burden on patients. Thus, it is necessary to identify the risk factors to prevent CRKP colonization and infections.

Molecular typing of bacterial isolates is the key strategy to identify clusters that are due to the transmission of clonal strains. Multilocus sequence typing (MLST), the repetitive sequence-based PCR Diversi Lab system and pulsed-field gel electrophoresis (PFGE) are good genotyping approaches, but these techniques remain time-consuming with a substantial cost. Rapid methods for molecular typing in colonization or infections with pathogens can not only provide basis for preventing cloning spread but also timely treatment. Therefore, quick methods that can be easily integrated into the routine work flow and do not cause increased costs are important (Sauget et al., 2017). Recently, matrix-assisted laser desorption ionization-time of flight mass spectrometry (MALDI-TOF MS) has been used as a simple tool for typing in infections with bacteria such as *Enterobacter cloacae* (Khennouchi et al., 2015). But other researcher do not recommend MALDI-TOF-based typing as a bacterial typing method given the heterogeneity in comparison to genotyping (Sachse et al., 2014). Thus, the application performance of MALDI-TOF MS as a clustering analysis method is still controversial.

Here, we set out to conduct a study for CRKP in Xiamen, a southern area in China, and we considered the following objectives: (1) study the epidemiology and risk factors for CRKP colonization and infections in this area, (2) evaluate the application performance of MALDI-TOF MS in clustering CRKP.

## Materials and Methods

### Patients and Settings

With the intent of examining prevalence, the background of the patients and the risk factors of CRKP acquisition (colonization and infection), we conducted a case-controlled study. A retrospective epidemiologic surveillance study of CRKP colonization and infections was conducted within a 1900-bed academic Medical Center in the southern area of China from 1 January 2015 to 31 January 2017. Either CRKP colonization or infections cases during the inpatients' stay period in hospital were classified as the case group. Patients who were negative for CRKP but positive for carbapenem-susceptible *Klebsiella pneumoniae* (CSKP) during their stay in hospital were used as the selection pool for the control group during the same study period. Exclusion criteria were community-acquired colonization and infections, missing key data, screening samples, and subsequent episodes in the same patient. The same exclusion criteria were applied to cases and controls.

CRKP cases were selected by a review of microbiological reports. All identified inpatients were initially eligible to participate, and their medical charts were reviewed. For inpatients with multiple episodes of colonization and infection with CRKP, only data relevant to the first episode were collected and analyzed. A colonization or infection case is defined according to CDC definitions of nosocomial infections (Garner et al., 1988).

The CSKP cases as control group were randomly selected from the same units where the inpatients isolated with CRKP during the study period. Records of the control participants were cross-referenced with microbiology results to ensure that they did not have any CRKP positive cultures. Controls whose records had insufficient information were replaced by other randomly selected controls. For inpatients with multiple episodes of infection with CSKP, only data relevant to the first episode were collected and analyzed. The age (±2 years) and sexes of the patients were matched to inpatients with CRKP colonization and infections, and the ratio for the CRKP:CSKP group was 1:2. We used age (±2 years) and sexes as the matching variables because both two are strong confunders and good candidates for direct mathing (Mansournia et al., 2018). We set a ratio of 1:2 in this study for two reasons: (1) concern for sufficient numbers in a stratified analysis; and (2) the increase in power given the expected prevalence of exposure among the controls (Hennessy et al., 1999).

Both case and control groups' data were collected from a database of hospital infection monitoring system. This database drew information from numerous sources, including patients' electronic health record, laboratory, microbiology, and medication administration records.

For identifying possible risk factors of CRKP colonization and infections, patients' demographic characteristics and medical conditions were collected from the electronic sources mentioned above by comparing the CRKP and CSKP groups.

This study was approved by the local Ethics Committee of The First Affiliated Hospital of Xiamen University and complied with the Declaration of Helsinki (2008). Written and informed consent was obtained from all participants.

### Definition of CRKP

A CRKP case was defined as the first clinical *Klebsiella pneumoniae* positive culture from inpatient with one or more of the following criteria, minimum inhibitory concentrations (MICs) for meropenem/imipenem ≥4 mg/L, MICs for ertapenem ≥2 mg/L according to the CLSI guidelines (CLSI, 2020).

### Microbiological Investigations

Species identification was performed with the Vitek 2 Compact automatic microbial analyzer (BioMerieux, Marcy—l'Etoile, France) and confirmed by matrix-assisted laser desorption/ionization time-of-flight mass spectrometry (MALDI-TOF MS; BioMerieux, Marcy-l'Etoile, France).

### Molecular Detection

Multiplex polymerase chain reactions (PCRs) were used to detect the presence of carbapenemase genes (*bla*_NDM_, *bla*_KPC_, *bla*_IMP_, and *bla*_VIM_). PCR products were sequenced, and the nucleotide and deduced protein sequences were analyzed with software programs that were available from the National Center of Biotechnology Information (NCBI) website (www.ncbi.nlm.nih.gov).

### Antimicrobial Susceptibility Testing

With regard the antimicrobial susceptibility test, MICs of ceftazidime, cefepime, cefotaxime, ceftriaxone, piperacillin/tazobactam, meropenem, ertapenem, imipenem, aztreonam, amikacin, gentamycin, tobramycin, ciprofloxacin, levofloxacin, trimethopri-sulfamethoxazole, and tigecycline were determined with the Vitek 2 Compact automatic microbial analyzer (BioMrieux, Marcy-l'Etoile, France) according to the Clinical and Laboratory Standards Institute (CLSI) guidelines. In addition, MICs of ertapenem, imipenem, meropenem and colistin-polymyxin-B were determined using E-test strips (BioMerieux, Marcy-l'Etoile, France) according to the manufacturer's instructions. Ertapenem, imipenem and meropenem MICs were interpreted according to the CLSI guidelines. The interpretive criteria for colistin-polymyxin-B was based on the breakpoints of EUCAST. And the interpretive criteria for tigecycline was based on the breakpoints of the Food and Drug Administration (FDA).

### Identification and Clustering of *Klebsiella Pneumoniae* Using MALDI-TOF MS

The *Klebsiella Pneumoniae* isolates were plated on Columbia blood agar (bioMérieux, Marcy l'Étoile, France) and incubated for 18 h to 24 h at 37°C. Isolated colonies of each strain were selected and used for MALDI-TOF MS identification using the MALDI-TOF MS (BioMerieux, Marcy-l'Etoile, France), as previously described (Rodel et al., 2019). The obtained spectra were manually selected in the spectra mode of SARAMIS Premium software (BioMerieux, Marcy-l'Etoile, France). Cluster analysis were performed by spectra compared to each other in SARAMIS RUO database according to the manufacturer's instructions (Vitek MS Plus SARAMIS Premium user manual, BioMerieux, Marcy-l'Etoile, France). Consensus spectra were analyzed with a single link agglomerative clustering algorithm, applying the relative taxonomy analysis tool of SARAMIS premium software to show the resulting dendrogram with differences and similarities in relative terms (percent matching masses). As a standard setting, the mass signal intensity was not considered in the cluster analysis. According to the type assignment, we defined a cut-off value was >75% similarity (Meng et al., 2019).

### Typing of *Klebsiella Pneumoniae* Using Pulse-Field Gel Electrophoresis (PFGE)

The 1 day, standardized PFGE protocol (Han et al., 2013) was used for all CRKP isolates during the study periods. Cell suspensions were placed in polystyrene tubes (Falcon; 12 × 75 mm), and their optical densities were adjusted to 3.8–4.0 by a Densimat photometer (BioMérieux, Marcy l'Etoile, France). Slices of CRKP agarose plugs were digested using 50 U of XbaI (TaKaRa Bio, Dalian, China) per slice for 4 h at 37°C, and electrophoresis was performed using a CHEF-DRIII system (Bio-Rad Laboratories, Hercules, CA, USA). Electrophoresis was conducted with a switch time of 6 to 36 s for 18.5 h, and images were captured using a Gel Doc 2000 system (Bio-Rad) and converted to TIFF files which were analyzed by BioNumerics version 5.1 software (Applied Maths, Kortrijk, Belgium). A similarity analysis of the PFGE patterns was performed by calculating the Dice coefficients (S_D_) and clustering was performed using the unweighted-pair group method with average linkages (UPGMA).

### Statistical Analysis

CRKP colonization and infections incidence was reported as the number of CRKP cases per 100,000 hospital patient-days. Descriptive statistics were used to summarize the clinical and epidemiologic characteristics of CRKP colonization and infections. Continuous variables were presented as medians with the range or interquartile range. For categorical variables, the percentage of patients or isolates in each category was calculated. The Chi-square test were used to compare categorical variables. The Mann-Whitney U-test was used to compare continuous variables. To identify risk factors for isolating CRKP, the Chi-square test were performed. Factors showing *p* < 0.05, were considered candidate predictors that were significantly related to CRKP isolation and were extracted; following which, multivariate analysis was performed for these factors using the Logistic Regression model. The discriminatory power of each typing method was assessed using Simpson's index of diversity (SID), calculating the probability that two unrelated strains sampled from the test population will be placed into different typing groups (Hunter and Gaston, 1988), and the 95% confidence intervals (CI) of the SID values were calculated as described previously (Grundmann et al., 2001). The quantitative concordance between typing methods was analyzed by using adjusted Randand Wallace coefficients (Carrico et al., 2006). All analyses were performed using the IBM SPSS statistical software package version 25 (IBM Corp, Armonk, NY, USA).

## Results

### Prevalence of CRKP Colonization and Infections

CRKP colonization and infections incidence during 1 January 2015 and 31 January 2017 was 2.7 (35/1,319,427) per 100,000 patient-days. During 1,319,427 patient-days, we found that 2,875 patients with *Enterobacteriaceae* isolates were obtained, and 36 patients with CRKP colonization and infections were eligible for screening in this study. After application of the exclusion criteria, 35 inpatients were included. Five of 35 patients isolating CRKP had infections. All the five were bloodstream infections, all were cured. The characteristics of the inpatients are shown in Table 1 and included 27 males and 8 females. The median age was 73 years (range 0–91 years).

**Table 1:** Comparison with patients' characteristics between CRKP and CSKP groups.

**Characteristic^**a**^**	**CRKP group^**b**^** **(*n* = 35) *n*, %**	**CSKP group^**b**^** **(*n* = 70) *n*, %**	***p*-value**
**Health care exposure during prior year**			
Acute care hospitalization	5 (14.3)	9 (12.9)	0.839
Dialysis	1 (2.9)	2 (2.9)	1.000
Resident of a long-term-care facility	6 (17.1)	14 (20.0)	0.725
Transfer to ICU within 30 days	6 (17.1)	5 (7.1)	0.115
Receipt of corticosteroids	4 (11.4)	9 (12.9)	0.834
**Underlying conditions**			
One or more underlying conditions	17 (48.6)	16 (22.9)	0.007
Cancer^c^	4 (11.4)	14 (20.0)	0.272
Diabetes mellitus	8 (22.9)	16 (22.9)	1.000
Heart diseases^d^	4 (11.4)	6 (8.6)	0.638
Hypertension	11 (31.4)	29(41.4)	0.320
Liver diseases^e^	7 (20.0)	14 (20.0)	1.000
Neurological diseases^f^	5 (14.3)	20 (28.6)	0.105
Pulmonary diseases^g^	28 (80.0)	25 (35.7)	<0.001
Renal diseases^h^	8 (22.9)	16 (22.9)	1.000
CCI score [Median (IQR)]	2.0 (4.0)	2.0 (4.0)	1.000
CCI ≥ 3	15 (42.9)	31 (44.3)	0.889
Smoking history	6 (17.1)	4 (5.7)	0.060
**Indwelling devices prior to culture**			
Central venous catheter	15 (42.9)	20 (28.6)	0.143
Gastric tube	24 (68.6)	21 (30.0)	<0.001
Tracheal cannula	5 (14.3)	12 (17.4)	0.708
Tracheotomy	10 (28.6)	10 (14.3)	0.079
Urinary catheter	20 (57.1)	36 (51.4)	0.580
**Laboratory findings**			
**White blood cells/mm**^**3**^			
Median (IQR)	12,350 (3, 900)	9,790 (6,853)	0.054
**Subgroup**			
<4,000	0 (0.0)	6 (8.6)	0.074
>10,000	24 (68.6)	39 (55.7)	0.205
C-reactive protein > 10 mg/liter	16 (45.7)	39 (55.7)	0.333
**Procalcitonin**			
0.5 to 2 ng/ml	5 (14.3)	20 (28.6)	0.105
>2 ng/ml	10 (28.6)	18 (25.7)	0.755
Use of proton pump inhibitors	8 (22.9)	18 (25.7)	0.749
Antifungal agents	8 (22.9)	5 (7.1)	0.021
**Antimicrobial use prior to culture within 30 days**			
One or more Antimicrobial uses	34 (97.1)	33 (47.1)	<0.001
Third- or fourth-generation cephalosporin use	6 (17.1)	15 (21.4)	0.605
Carbapenem use	16 (45.7)	5 (7.1)	<0.001
Quinolone use	14 (40.0)	6 (8.6)	<0.001
**Specimen isolating** ***Klebsiella pneumoniae***			
Respiratory specimen	13 (37.1)	31 (44.3)	0.484
Urine	9 (25.7)	16 (22.9)	0.746
Blood	6 (17.1)	13 (18.6)	0.858
Ascites	2 (5.7)	2 (2.9)	0.471
Bile	1 (2.9)	3 (4.3)	0.718
Skin	1 (2.9)	2 (2.9)	1.000
Others	3 (8.6)	3 (4.3)	0.372
Length of stay [Median (IQR)]^i^	34 (38.0)	16 (20.0)	<0.001
**Discharge disposition**			
Recovery	7 (20.0)	13 (18.6)	
Improvement	18 (51.4)	39 (55.7)	0.375
Patients transfer to other hospital	0 (0.0)	1 (1.4)	
Functional status deterioration	9 (25.7)	16 (22.9)	
In-hospital mortality	1 (2.9)	1 (1.4)	0.015
Medical expense for admission^i^ (Mean ± SD, RMB)	107,472.27 ± 110,564.67	60,738.59 ± 72,925.18	

*CRKP, carbapenem-resistant Klebsiella pneumoniae; CSKP, carbapem-sensitive Klebsiella pneumoniae; CCI, Charlson comorbidity index*.

a *IQR, interquartile range; ICU, intensive care unit*.

b *Data are presented as the number/total number (%), unless otherwise indicated*.

c *Cancer includes malignancy of the lung, digestive tract, gynecology, hematological system, and neurological system*.

d *Heart diseases include congestive heart failure, coronary heart disease, valve replacement, and congenital heart disease*.

e *Liver diseases included cirrhosis, hepatitis, liver abscess, hepar adiposum (i.e., fatty liver), and hepatic injury*.

f *Neurological diseases include stroke, transient ischemic attack, cerebral palsy, and meningitis*.

g *Pulmonary diseases included chronic obstructive pulmonary disease (COPD), asthma, interstitial lung disease, history of pneumonia and tuberculosis, emphysema, respiratory failure, and infection*.

h *Renal diseases include azotemia and chronic kidney disease*.

i *Only patients admitted to hospital were evaluated*.

### Clinical and Microbiological Characteristics in CRKP Inpatients

We found that 25.7% (9/35) of inpatients had functional status deterioration seen in Table 1. One patient in ICU died within 30 days of admission that was not due to that of a bloodstream infection, but of multiple organ failure caused by cancer, the same reason as the one patient died in CSKP group. CRKP group patients had higher medical expense than those among CSKP group (as shown) in Table 1 (*p* = 0.015). With regard the antimicrobial susceptibility test, colistin-polymyxin-B, and tigecycline retained excellent activity, with a susceptibility rate of more than 97%. Trimethopri-sulfamethoxazole remained quite susceptible, with susceptibility rate of 57.1%. All isolates of CRKP were detected producing KPC-2 carbapenemase. Further, no CRKP was detected producing two or more gene types of carbapenemase.

### Analysis of Risk Factors for Patients Isolating CRKP

The results of univariate analysis using the Chi-square test in patients with CRKP are shown in Table 1. Eight parameters were associated with patients isolating CRKP, namely one or more underlying conditions (*p* = 0.007), pulmonary diseases (*p* < 0.001), gastric tube (*p* < 0.001), antifungal agents (*p* = 0.021), one or more antimicrobial use prior to culture within 30 days (*p* < 0.001), carbapenem use (*p* < 0.001), quinolone use (*p* < 0.001) and length of stay (*p* < 0.001).

Multivariate logistic regression analysis was applied to analyze the prognostic significance of these eight factors, revealing that one or more underlying conditions (*p* = 0.031, odds ratio [OR]: 3.991, 95% confidence interval [CI]: 1.132–14.068), pulmonary diseases (*p* = 0.007, odds ratio [OR]: 5.293, 95% confidence interval [CI]: 1.590–17.618), one or more antimicrobial use prior to culture within 30 days (*p* = 0.009, odds ratio [OR]: 17.358, 95% confidence interval [CI]: 2.051–146.931) and carbapenem use (*p* = 0.018, odds ratio [OR]: 5.118, 95% confidence interval [CI]: 1.321–19.829) were indeed independent risk factors for patients isolating CRKP. Four different clusters of 35 KPC-2-producing CRKP isolates were identified by PFGE and MALDI-TOF MS. Cluster I, II, and III were mainly isolated from geriatrics and respiratory wards. Cluster IV was mainly isolated from pediatrics and icu departments.

### Clonal Typing KPC-2 Producing CRKP by PFGE

**Figure 1:**
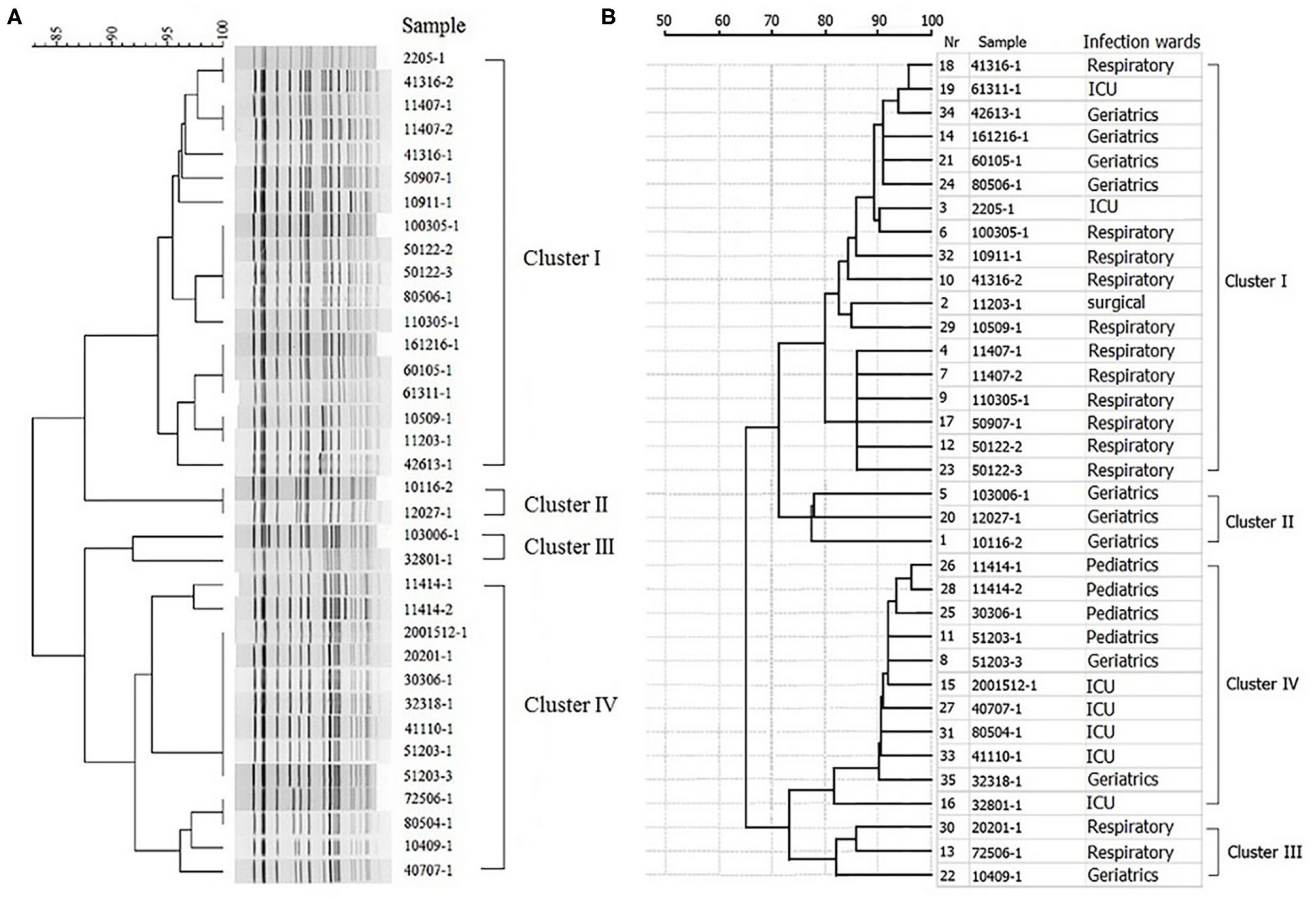
Hierarchical clustering of KPC-2-producing carbapenem-resistant *Klebsiella pneumoniae* isolates by PFGE and MALDI-TOF MS. **(A)** Hierarchical cluster analysis provided by PFGE. **(B)** Hierarchical cluster analysis provided by MALDI-TOF MS.

The PFGE system identified four different clusters of 35 KPC-2-producing CRKP isolates (Figure 1A). All indistinguishable isolates in four clusters presented an average genomic similarity ratio of >90.0%. The four clusters were significantly different from each other in the percentage of similarity.

### Clustering CRKP Isolates Using MALDI-TOF MS

All the 35 CRKP isolates were correctly identified at the species level by MALDI-TOF MS. The hierarchical clustering of MALDI-TOF peak profiles identified four different clusters, substantially interchangeable with those obtained with the PFGE system (Figure 1B). The statistical analysis of the data showed that the PFGE system (Simpson's index, 0.608; 95% CI, 0.512–0.705) and MALDI-TOF MS system (Simpson's index, 0.640; 95% CI, 0.532–0.748) provided similar results, with a good concordance between the two methods (adjusted Rand's coefficient, 0.846) and a high probability of MALDI-TOF MS to predict PFGE results (Wallace coefficient, 0.908).

## Discussion

This present retrospective case-controlled study assessed potential risk factors for the development of colonization and infections by CRKP in hospitalized patients. In this study, it demonstrates that inpatients with one or more underlying conditions, especially pulmonary diseases, and antimicrobial use prior to culture within 30 days, particularly carbapenem use, were risk factors for CRKP acquisition. And four different clusters of KPC-2-producing CRKP isolates were identified. Cluster I, II, and III were mainly isolated from geriatrics and respiratory wards. Patients with underlying conditions, such as pulmonary diseases, often visit an outpatient clinic or transfer from icu to respiratory ward, or transfer between the two, even from one hospital to another hospital, and they are subsequently exposed to additional health care and antimicrobials, which are among the most prominent risks (Gupta et al., 2011). These patients could have poor functional status and severe clinical symptoms, which not only places them at a greater risk of an infection caused by CRKP but also results in higher medical expense. Our study demonstrated that medical expense for admission of CRKP groups were almost double higher than those of CSKP group (107,472 vs. 60,739 RMB, *p* = 0.015).

Among the four classes of β-lactamases defined by the Ambler classification system, the KPC β-lactamase, in Bush group 2f, belongs to Class A. Yigit et al. (2001) first reported KPC β-lactamases in *Klebsiella pneumoniae* strains isolated from a patient in North Carolina in the United States in 2001. After that, the KPC-producing organisms had being reported globally (Villegas et al., 2006; Wiener-Well et al., 2010; Canton et al., 2012; Mojica et al., 2012; Cuzon et al., 2013; Asai et al., 2018; Kim et al., 2018). Since in 2015, Biberg et al. (2015) reported KPC-2-producing *Klebsiella pneumoniae* in the Midwest region of Brazil, the rapid increase and dissemination of KPC-2, the primary type of β-lactamases, in CRKP from many areas, has become a significant public health challenge in the whole word (Gaiarsa et al., 2015). In this study, all CRKP isolates were detected with KPC-2 carbapenemase. The *bla*_KPC−2_ is the mainstream gene of CRKP in our geographic area of analysis.

Bacterial typing is an important method to identify the route of pathogen transmission. Currently, the main method for bacterial typing is the time-consuming and expensive molecular biology technique like Pulsed Field Gel Electrophoresis (PFGE) or Multilocus sequence typing (MLST). Nevertheless, with the application to cultured microorganism identification, matrix-assisted laser desorption/ionization time-of-flight mass spectrometry MS (MALDI-TOF MS) presents incomparable advantages. However, as a new method of bacteria clustering, the application performance of mass spectrometry is controversial. Some studies reported that MALDI-TOF MS could be a good bacterial typing method in several kinds of bacteria, such as extended-Spectrum-β-Lactamase- and *armA* methyltransferase-producing *Enterobacter cloacae* clinical isolates, methicillin-resistant *Staphylococcus aureus, Acinetobacter baumannii, Serratia marcescens*, and *Citrobacter freundii* (Mencacci et al., 2013; Khennouchi et al., 2015; Steensels et al., 2017; Rodel et al., 2019). But, Jiang et al. (2019) employed 44 CRKP isolates of 15 STs covering divere carbapenemases and they demonstrated that MALDI-TOF MS had a lower predictive power than PFGE. And Sachse et al. (2014) did not recommend MALDI-TOF-based typing as a bacterial typing method given the heterogeneity in comparison to genotyping.

In this study, all 35 CRKP isolates were correctly identified at the species level by MALDI-TOF MS. The hierarchical clustering of MALDI-TOF peak profiles identified four different clusters, substantially interchangeable with those obtained with the PFGE system. The statistical analysis of the data showed that the PFGE system and MALDI-TOF MS system provided similar results, with a good concordance between the two methods and a high probability of MALDI-TOF MS to predict PFGE results. Since rapid microorganism identification using MADI-TOF MS not only can lead to more effective antimicrobial use and reduced patient care costs (Galar et al., 2012; Tan et al., 2012; Huang et al., 2013; Perez et al., 2013), but also include the high through put, low reagent costs and ease of use, the usage of MALDI-TOF MS in clustering the CRKP of epidemic KPC-2 type was an agreeable practice and the subsequent clinical application would be meaningful to both hospital infection control and patients. It could be one of the choices to rapidly reveal the routes of transmission of infectious diseases. However, because of the small size of sample, further studies are needed to confirm our observations.

There were three limitations of this study. Firstly, information on the clinical characteristics and outcomes could not be completely acquired because of the limitations that are inherent in a retrospective clinical study. Second, this is a retrospective study with a relatively small study population. Furthermore, this study was a case-controlled design in which the level of risk factors were not equal to the expected level commonly seen in the population.

## Conclusions

One or more underlying conditions, especially pulmonary diseases, and one or more antimicrobial use prior to culture within 30 days, particularly carbapenem use, are risk factors for CRKP acquisition. The *bla*_KPC−2_ is the mainstream gene of CRKP in our geographic area of analysis. As only simple sample preparation is needed and the results can be obtained in a short time, MALDI-TOF MS may be considered a probable alternative to PFGE in clustering KPC-2-producing CRKP.

## Data Availability Statement

The raw data supporting the conclusions of this article will be made available by the authors, without undue reservation.

## Ethics Statement

The studies involving human participants were reviewed and approved by the local Ethics Committee of The First Affiliated Hospital of Xiamen University. Written informed consent to participate in this study was provided by the participants' legal guardian/next of kin. Written informed consent was obtained from the individual(s), and minor(s)' legal guardian/next of kin, for the publication of any potentially identifiable images or data included in this article.

## Author Contributions

LF, HX, and XR designed the study. XLi, XM, and HZ analyzed data. LF and XLia drafted the manuscript. GH and XLia contributed to the final version of the manuscript. All authors contributed to the article and approved the submitted version.

## Conflict of Interest

The authors declare that the research was conducted in the absence of any commercial or financial relationships that could be construed as a potential conflict of interest.

## Abbreviations

CRKP: carbapenem-resistant *Klebsiella pneumoniae*
CSKP: carbapenem-susceptible *Klebsiella pneumonia*
MALDI-TOF MS: matrix-assisted laser desorption ionization-time of flight mass spectrometry
MLST: multilocus sequence typing
PFGE: pulsed-field gel electrophoresis
PCRs: multiplex polymerase chain reactions
NCBI: National Center of Biotechnology Information
FDA: Food and Drug Administration
MICs: minimum inhibitory concentrations
CLSI: the Clinical and Laboratory Standards Institute
IQR: interquartile range
ICU: intensive care unit
CCI: Charlson comorbidity index
COPD: pulmonary diseases included chronic obstructive pulmonary disease
OR: odds ratio
aOR: adjusted odds ratio
CI: confidence interval
SID: Simpson's index of diversity.
