# Supplementary material for: Epidemiology and Risk Factors for Carbapenem-Resistant Klebsiella Pneumoniae and Subsequent MALDI-TOF MS as a Tool to Cluster KPC-2-Producing Klebsiella Pneumoniae, a Retrospective Study
Source: Front Cell Infect Microbiol. 2020 Sep 14;10:462. doi: 10.3389/fcimb.2020.00462 (PMC7521130; doi:10.3389/fcimb.2020.00462)
Supplement: Supplementary file 1 [file Table_1.doc]

**Table S1. Comparison of antibiotic susceptibility between CRKP and CSKP** groups.

| **Antimicrobial agent** | **CRKP group n = 35 (%)** | **CSKP group n = 70 (%)** | ***p*-value** |
| --- | --- | --- | --- |
| Meropenem | 0 (0.0) | 70 (100.0) | <0.001 |
| Imipenem | 2 (5.7) | 70(100.0) | <0.001 |
| Ertapenem | 0 (0.0) | 70 (100.0) | <0.001 |
| Cefepime | 2 (5.7) | 62 (88.6) | <0.001 |
| Ceftazidime | 0 (0.0) | 59 (84.3) | <0.001 |
| [Cefotaxime](app:ds:cefotaxime) | 0 (0.0) | 49 (70.0) | <0.001 |
| [Ceftriaxone](app:ds:ceftriaxone) | 0 (0.0) | 49 (70.0) | <0.001 |
| Piperacillin/Tazobactam | 0 (0.0) | 60 (85.7) | <0.001 |
| Amikacin | 7 (20.0) | 69 (98.6) | <0.001 |
| [Tobramycin](app:ds:tobramycin) | 2 (5.7) | 57 (81.4) | <0.001 |
| Gentamycin | 3 (8.6) | 67 (95.7) | <0.001 |
| Ciprofloxacin | 5 (14.3) | 54 (77.1) | <0.001 |
| [Levofloxacin](app:ds:levofloxacin) | 5 (14.3) | 55 (78.6) | <0.001 |
| Trimethopri-sulfamethoxazole | 20 (57.1) | 47 (67.1) | = 0.315 |
| [Aztreonam](app:ds:aztreonam) | 3 (8.6) | 59 (84.3) | <0.001 |
| [Tigecycline](app:ds:Tigecycline) | 34 (97.1) | 70 (100.0) | = 0.155 |
| Clistin-polymyxin-B | 35 (100.0) | 70 (100.0) | = 1.000 |

CRKP, carbapenem-resistant *Klebsiella pneumoniae*; CSKP, carbapem-sensitive *Klebsiella pneumoniae*.
