# Supplementary material for: Epidemiology and Risk Factors for Carbapenem-Resistant Klebsiella Pneumoniae and Subsequent MALDI-TOF MS as a Tool to Cluster KPC-2-Producing Klebsiella Pneumoniae, a Retrospective Study
Source: Front Cell Infect Microbiol. 2020 Sep 14;10:462. doi: 10.3389/fcimb.2020.00462 (PMC7521130; doi:10.3389/fcimb.2020.00462)
Supplement: Supplementary file 2 [file Table_2.doc]

**Table S2. Risk factors for patients isolating CRKP**.

| **Variables** | **Univariate analysis** |  | **Multi-variate analysis** |  |
| --- | --- | --- | --- | --- |
|  | **OR (95% CI)** |  | **aOR (95% CI)** | ***p-*value** |
| **One or more underlying conditions** | 3.187 (1.340-7.581) |  | 3.991 (1.132-14.068) | 0.031 |
| **Pulmonary diseases** | 7.200 (2.752-18.837) |  | 5.293 (1.590-17.618) | 0.007 |
| **Gastric tube** | 5.091 (2.116-12.247) |  |  |  |
| **Antifungal agents** | 3.852 (1.155-12.841) |  |  |  |
| **One or more antimicrobial uses prior to culture within 30 days** | 38.121 (4.941-294.116) |  | 17.358 (2.051-146.931) | 0.009 |
| **Carbapenem use** | 10.947 (3.548-33.781) |  | 5.118 (1.321-19.829) | 0.018 |
| [**Quinolone**](app:ds:quinolone) **use** | 7.111 (2.425-20.854) |  |  |  |
| **Length of stay** | 2.292 (1.332-3.944) |  |  |  |

OR, odds ratio; aOR, adjusted odds ratio; CI, confidence interval.
